# The *MdmiR156n* Regulates Drought Tolerance and Flavonoid Synthesis in Apple Calli and *Arabidopsis*

**DOI:** 10.3390/ijms24076049

**Published:** 2023-03-23

**Authors:** Guo Chen, Yaping Wang, Xueli Liu, Siyue Duan, Shenghui Jiang, Jun Zhu, Yugang Zhang, Hongmin Hou

**Affiliations:** 1 College of Horticulture, Qingdao Agricultural University, Qingdao 266109, China; 2 Engineering Laboratory of Genetic Improvement of Horticultural Crops of Shandong Province, Qingdao Agricultural University, Qingdao 266109, China

**Keywords:** miR156, apple, flavonoid synthesis, drought tolerance

## Abstract

Drought is the major abiotic stress that limits apple productivity and quality. To date, many important and divergent regulatory functions of miR156/SBP genes in plant growth and development have been well understood. However, little is known about the role of apple miR156 in response to abiotic stress. To better understand the functions of *MdmiR156* in abiotic stress tolerance, we constructed the overexpression (OE) and short tandem target mimic (STTM) vector of *MdmiR156n* and performed its functional analysis through the characterization of transgenic apple calli and *Arabidopsis thaliana* plants. In this study, *MdmiR156n* overexpression significantly increased the length of primary roots and the number of lateral roots in transgenic Arabidopsis plants under drought stress. In addition, *MdmiR156n* transgenic Arabidopsis and apple calli had a lower electrolyte leakage rate and less cell membrane damage than WT and STTM156 after drought stress. Further studies showed that *MdmiR156n* overexpression promoted the accumulation of flavonoids and scavenging of reactive oxygen species (ROS) under drought conditions in transgenic apple calli and *A. thaliana* plants. Taken together, overexpression *MdmiR156n* enhances drought tolerance by regulating flavonoid synthesis and ROS signaling cascades in apple calli and *A. thaliana*.

## 1. Introduction

Plants often experience unexpected environmental changes, including drought, salinity, and heat and cold stress, which severely affect their growth and development and greatly reduce the quality and yield of their crop. We all know that plants need adequate water for growth and development. However, with global warming, drought has become one of the most severe and common environmental factors limiting crop production worldwide. In order to adapt to the environment, plants have evolved a range of response mechanisms to resist drought stress. To date, many drought-stress-related genes and pathways have been isolated and identified in different plant species.

MicroRNAs are non-coding small RNA molecules with 20–24 nucleotides that play crucial roles in transcriptional and post-transcriptional gene regulation in plants and animals [1]. They can cause target gene degradation or translation inhibition by transcriptional cleavage and/or translational repression [2]. As a conserved and ancient miRNA family, microRNA156s (miR156s) have been found in bracken, mosses, and higher plants [3]. Some of them have been reported to be involved in diverse processes of plant growth and development by constituting a class of miR156/SPL modules. For example, the miR156/SPL model can control shoot development [4], leaf development [5], tiller number and plant architecture [6,7], lateral root formation [8,9], and the juvenile-to-adult phase transitions [10,11,12].

To date, many important and divergent regulatory functions of miR156/SBP genes in plant growth and development have been well understood. However, only a small number of these genes have been shown to play a role in the response to abiotic stresses. For example, in Arabidopsis, it has been reported that miR156 increased the heat and freezing tolerance by inhibiting the expression of *AtSPLs* [13,14]. Similarly, miR156 has recently been implicated in improving the heat, salt, and drought tolerance of alfalfa by suppressing *SPL* gene expression [15,16,17,18,19]. The overexpression of *ZmmiR156* from maize improved the tolerance to drought and salt in transgenic tobacco [20]. In addition, *OsmiR156* enhances cold stress tolerance in rice through down-regulation of *OsSPL3* [21]. *MiR156* positively regulates plant tolerance to Cd stress by regulating Cd uptake/transport gene expression [22]. Furthermore, when plants experience continuous environmental stress, reactive oxygen species (ROS) will accumulate excessively in plant cells, causing lipid hyperoxidation and eventually cell death [23,24]. As an ancient, specialized group of secondary metabolites in plants, flavonoids have been extensively reported to participate in plant–environment interactions owing to their strong antioxidant and scavenging capacity for ROS [25,26]. Recently, knowledge concerning the functions of *miRNA156s* in flavonoid synthesis has begun to accumulate. Overexpression of *AtmiRNA156* promotes the accumulation of anthocyanins in the lateral branch and inflorescence–stem junction of Arabidopsis [27]. Overexpression of *VcMIR156a* in tomato *(Solanum lycopersicum*) enhanced anthocyanin biosynthesis in the stem by altering pigment-associated gene expression [28]. The miR156 regulates anthocyanin biosynthesis through SPL targets and other microRNAs in poplar [29]. 

Previous studies have shown that miR156 plays an important role in combating environmental stress and flavonoid synthesis. Unfortunately, these studies mainly focused on model plant *A. thaliana* and herbaceous plants such as rice, alfalfa, and maize, while there were few studies on the regulatory mechanism of miR156/SBP in woody plants. Apple (*Malus domestica*) is an economically important fruit tree that grows widely throughout the world. However, as a perennial fruit tree, apple trees are subject to a variety of biotic and abiotic stresses due to their inability to escape harsh conditions during their growth and development. Among these, drought is one of the main factors affecting apple yield and quality. In this investigation, the overexpression of *MdmiR156n* remarkably decreased malondialdehyde (MDA) and superoxide anion (O_2_^−^) accumulation and increased superoxide dismutase (SOD) content in transgenic *Arabidopsis* and ‘Orin’ calli under drought treatment. Furthermore, overexpression of *MdmiR156n* may improve drought resistance by regulating flavonoid synthesis. Taken together, our study uncovered the biological functions of apple *MdmiR156n* in drought stress and flavonoid synthesis, thus providing a new basis for apple resistance and quality improvement.

## 2. Results

### 2.1. Overexpression of Mdmir156n Enhanced the Drought Tolerance in Transgenic ‘Orin’ Calli

**Figure 1:**
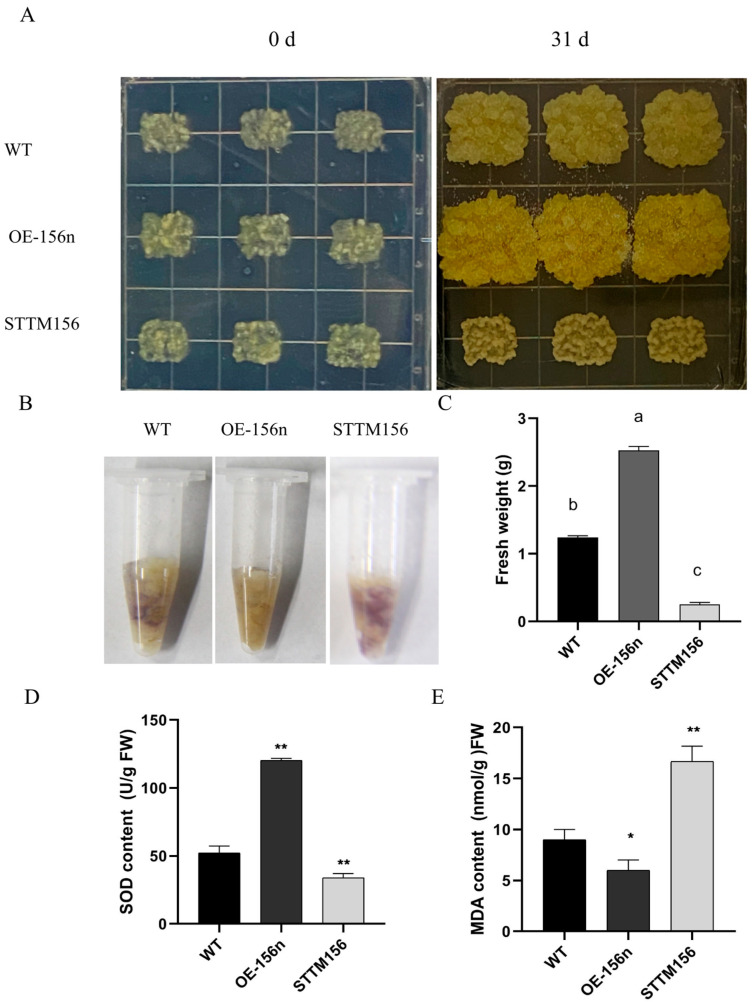
*Mdmir156n* is involved in drought stress in apple calli: (**A**) WT, OE-156n, and STTM156 apple calli cultured in medium with 6% PEG. (**B**) Histochemical staining of NBT for O_2_^−^ accumulation in apple calli after PEG treatment for 0.5 h. (**C**) The weights of WT, OE-156n, and STTM156 apple calli lines after 6% PEG treatment for 31 d. Different letters indicate statistical differences (*p* < 0.05). (**D**) Measurement of SOD activities. (**E**) Measurement of MDA content. Asterisks indicate statistically significant differences (* *p* < 0.05, ** *p* < 0.01, one-way ANOVA). Values are means SD of three independent biological replicates.

Rooted apple seedlings from tissue culture were grown using hydroponics in the presence of polyethylene glycol (PEG)-6000 to perform a drought-stress simulation. *MdmiR156* was significantly induced by 6% PEG, suggesting that *MdmiR156* may be associated with drought stress responses (Figure S1). To understand the biological functions of miR156 in apple responses to drought stresses, precursor genes of *MdmiR156n* were cloned from an apple rootstock ‘e zhen-5’ with the primer pair 156n-F and 156n-R (Table S1, Figure S2A). Then, the vectors of *MdmiR156n* overexpression and STTM156 were constructed and transformed into apple ‘Orin’ calli. The OE-156n and STTM-156n transgenic lines were obtained by kanamycin screening and qRT-PCR identification (Supplementary Figure S2). The two lines (OE-156n-3 and STTM156-7) with the best performance and the highest or lowest levels of expression were selected for further study (Figure S2). The same weight of wild-type (WT) and transgenic apple ‘Orin’ calli (OE-156n and STTM156) was placed on Murashige and Skoog (MS) medium with 6% PEG in the same Petri dish. However, their growth was markedly different after 31 days of drought treatment. Compared to WT, OE-miR156n could grow normally with a deep yellow color and was least affected by stress. In contrast, STTM156 suffered the most serious injuries causing its death (Figure 1A). Similarly, fresh weight measurements showed that OE-miR156n was over two-fold heavier than WT, while STTM156 was significantly lighter than WT. In addition, the levels of superoxide anion radicals (O_2_^−^) in these ‘Orin’ calli after 6% PEG treatment were examined using nitrotetrazolium blue chloride (NBT) histochemical staining. It can be seen from the graphs that STTM156, WT and OE-156n stained with decreasing color depth, indicating that they were also subjected to decreasing oxidative stress injury (Figure 1C). Taken together it indicates that *MdmiR156n* overexpression can improve the tolerance of ‘Orin’ calli to drought stress. 

Drought stress enhances the production of reactive oxygen species (ROS) and causes oxidative damage to cell membranes. It is well known that antioxidant enzymes play a central role in ROS scavenging and plant defense. To further investigate the correlation between ROS reduction and antioxidant enzyme activities, the enzyme activities of SOD and MDA content were measured in WT and transgenic apple calli grown under the same osmotic stress conditions (Figure 1D,E). The results showed that there were significantly lower MDA levels and significantly higher SOD activity in OE-156n, compared to the WT. However, the MDA content and SOD activity in STTM156 were the opposite of those in OE-156. The above results indicated that overexpression of *Mdmir156n* enhanced the drought tolerance in transgenic ‘Orin’ calli.

### 2.2. Overexpression of Mdmir156n Enhanced the Drought Tolerance in Transgenic A. thaliana

**Figure 2:**
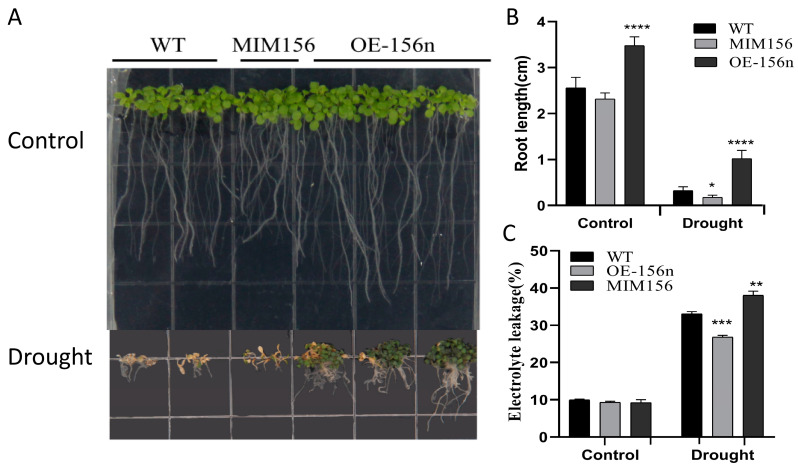
Analysis of the PEG-simulating drought tolerance of WT, OE-156n, and MIM156 transgenic *A. thaliana* seedlings. (**A**) Phenotypes of WT, OE-156n, and MIM156 transgenic lines under simulated drought stress. (**B**) Root length of WT, OE-156n, and MIM156 lines. (**C**) Relative electrolyte leakage rate of WT, OE-156n, and MIM156 lines. Values are means SD of three independent biological replicates. Asterisks indicate statistically significant differences (* *p* < 0.05, ** *p* < 0.01, *** *p* < 0.001 and **** *p* < 0.0001, one-way ANOVA).

To investigate the function of *MdmiR156n* in response to drought stress, we also heterologously transformed *Mdmir156n* into Arabidopsis. Then, WT, OE-156n, and MIM156 transgenic Arabidopsis seeds were sown on MS medium containing 6% PEG for simulating drought treatment (Figure 2). WT, OE-156n, and MIM156 seedlings all grew normally on MS medium. However, most WT and MIM156 plants started to turn yellow and died after 20 days on PEG-stressed medium, and only OE-156n seedlings could continue to grow (Figure 2A). Further, we found that the root length of OE-156n plants was significantly longer than that of WT and MIM156 plants in both normal MS culture and medium containing PEG (Figure 2B). Meanwhile, we measured changes in relative electrolyte leakage in the WT, OE-156n, and MIM156 seedlings to determine whether there was a correlation with the improved PEG stress tolerance of the OE-156n transgenic seedlings. The results showed that the relative electrolyte leakage of control seedlings grown on MS medium was not significantly different. However, the relative electrolyte leakage rate was significantly lower in OE-156n lines than in WT and MIM156 seedlings grown on medium with 6% PEG (Figure 2C).

**Figure 3:**
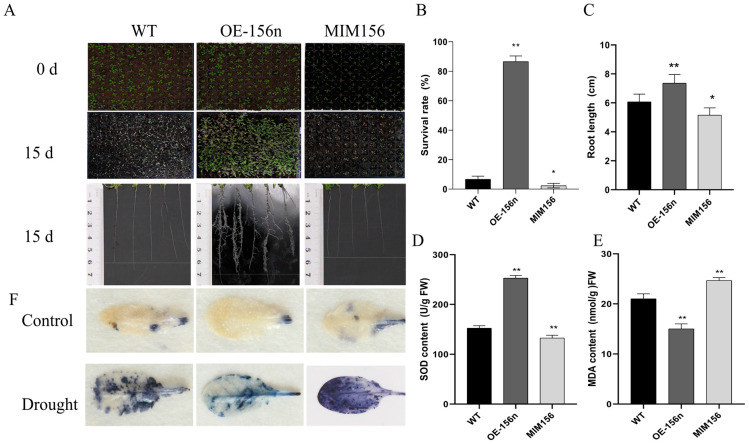
Overexpression of *Mdmir156n* enhanced the drought tolerance in transgenic *A. thaliana*: (**A**) Phenotypes of WT, OE-156n, and MIM156 lines after natural drought for 15 days. (**B**) Survival rates of WT, OE-156n, and MIM156 lines after re-watering for 3 d. (**C**) The root length of WT, OE-156n, and MIM156 lines after drought treatment for 15 days. (**D**) Measurement of SOD activities in WT, OE-156n, and MIM156 lines. (**E**) Measurement of MDA content in WT, OE-156n, and MIM156 lines. (**F**) Histochemical staining of NBT for O_2_^−^ accumulation in WT, OE-156n, and MIM156 lines after drought treatment for 15 d. Values are means SD of three independent biological replicates. Asterisks indicate statistically significant differences (* *p* < 0.05, ** *p* < 0.01, one-way ANOVA).

Further, seedling-aged 10-day-old WT and transgenic plants were subjected to prolonged natural drought treatment in a nutrient bowl. After 15 days of drought treatment, all WT and MIM156 plants exhibited severe water-loss-related symptoms and substantial wilt mortality (Figure 3A). In contrast, OE-156n transgenic plants exhibited more lateral roots and only slight yellowing under the same conditions, with a survival rate of more than 80% (Figure 3A,B). Histochemical staining of leaves with NBT showed that leaf O_2_^−^ accumulation was significantly lower in the OE-156n plants than in the WT and MIM156 lines after 1 week of drought treatment (Figure 3F). In addition, the OE-156n lines exhibited higher SOD activity and lower MDA content compared with WT and MIM156 transgenic plants (Figure 3D,E). Taken together, these results suggest that *MdmiR156n* heterologous expression enhanced the drought tolerance of Arabidopsis.

**Figure 4:**
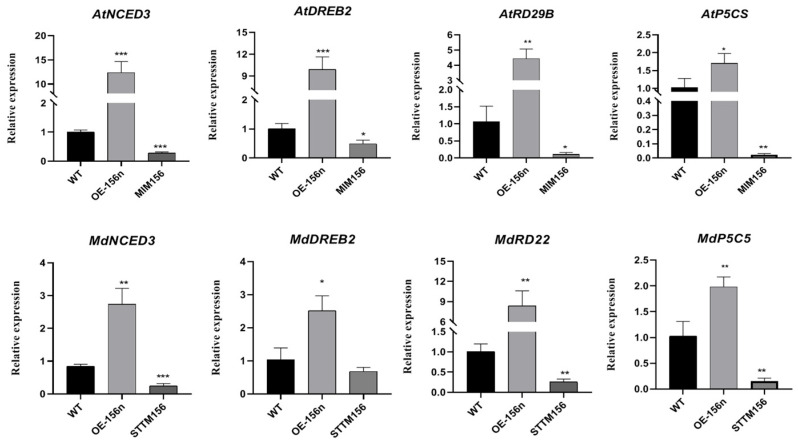
Expression analysis of stress-related genes in *Mdmir156n* transgenic A. thaliana. and apple calli under 6% PEG treatment. Values are means SD of three independent biological replicates. Asterisks indicate statistically significant differences (* *p* < 0.05, ** *p* < 0.01 and *** *p* < 0.001, one-way ANOVA).

In addition, to further understand the mechanism of *MdmiR156n* to improve drought resistance, we examined the expression of stress-related genes in transgenic Arabidopsis and apple calli by real-time quantitative PCR. Compared with WT, *AtNCED3*, *AtDREB2*, *AtRD9B*, and *AtP5CS* were upregulated in the OE-156n transgenic *A. thaliana* after drought stress. However, their expression pattern in the MIM156 line was opposite to that of the OE-156n. Similarly, the expression of *MdP5CS*, *MdRD22*, *MdDREB2*, and *MdNCED3* in OE-156n or STTM156n apple calli was significantly higher or lower than that of WT after drought treatment (Figure 4). In summary, we can speculate that *Mdmir156n* can improve drought resistance in apple and Arabidopsis by regulating the expression of downstream stress-related genes.

### 2.3. Mdmir156n Is Involved in the Drought-Induced Accumulation of Flavonoids and Anthocyanins in Transgenic A. thaliana under Natural Drought Conditions

**Figure 5:**
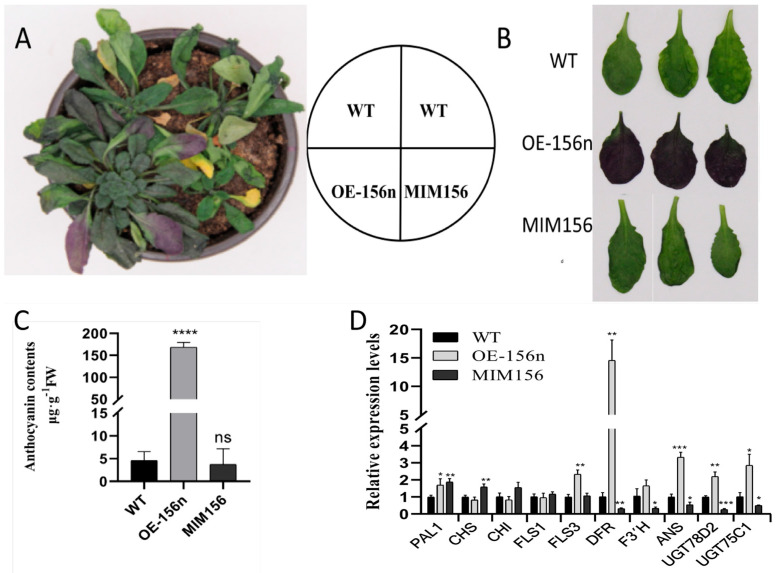
*Mdmir156n* is involved in the drought-induced accumulation of flavonoids and anthocyanins: (**A**) Phenotypes of WT, OE-156n, and MIM156 transgenic *A. thaliana* lines under drought treatment. (**B**) Anthocyanin accumulation on the back of leaves deprived of water for 10 days. (**C**) Anthocyanin contents in wild-type, OE-156n, and MIM156 lines under drought treatment. (**D**) Expression analysis of the flavonoid biosynthetic pathway genes in WT, OE-156n, and MIM156 lines. Values are means SD of three independent biological replicates. Asterisks indicate statistically significant differences (* *p* < 0.05, ** *p* < 0.01, *** *p* < 0.001, and **** *p* < 0.0001, ns: no significant difference, one-way ANOVA).

Previous studies demonstrated that drought can induce the accumulation of flavonoids and anthocyanins in plants [30,31]. To further evaluate the function of *MdmiR156n* in drought-induced flavonoid synthesis, the 20-day-old WT and transgenic seedlings (OE-156n and MIMI156) grown in the same pot were exposed to natural drought for 10 days. The OE-156n seedings showed significant drought resistance, while the WT and MIMI156 seedlings had withered yellow leaves and grew slower (Figure 5A). Notably, a large amount of anthocyanin accumulation appeared on the abaxial surface of the leaves of OE-156n plants (Figure 5B). Quantitative analysis showed that the anthocyanin content of OE-156n Arabidopsis was up to 150 μg.g^−1^, which was over 30-fold higher than that of MIM156 and WT lines (Figure 5C). We also measured the expression profiles of a number of the flavonoid biosynthetic pathway genes in WT and transgenic plants under drought treatments using quantitative real-time PCR (qRT-PCR) analysis. We found that the OE-156n lines had higher expression levels of ANS, DFR, F3H, UGT75C1, and UGT78D2. In particular, DFR transcript level in OE-156n was 10-fold greater than that of WT, whereas it was significantly lower in MIM156. In addition, the expression patterns of ANS, F3′H, UGT78D2, and UGT75C1 were identical to those of DFR in WT, OE-156n, and MIM156 (Figure 5D). Thus, we speculated that *Mdmir156n* may improve the plants drought resistance through the accumulation of flavonoids and anthocyanins.

### 2.4. Mdmir156n Is Involved in the Drought-Induced Accumulation of Flavonoids and Anthocyanins in Transgenic Apple Calli under Drought Conditions

**Figure 6:**
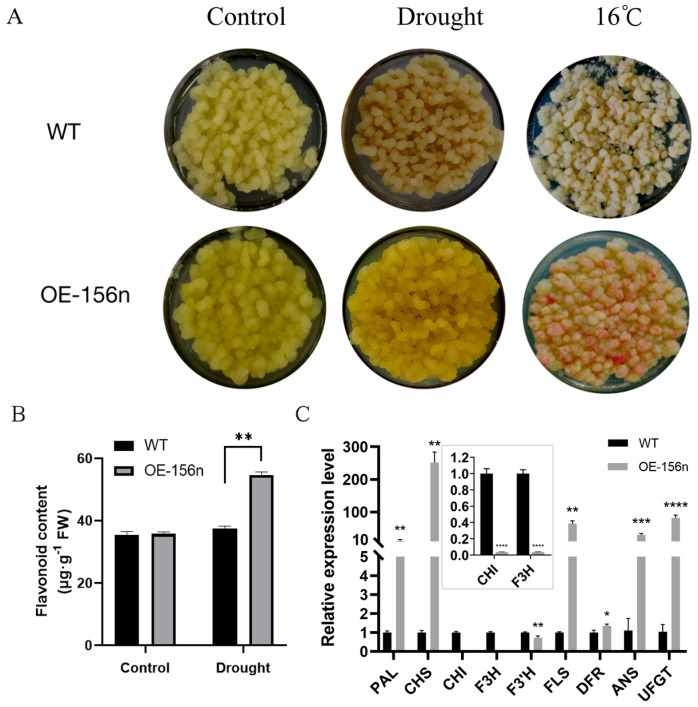
*Mdmir156n* is involved in the drought-induced accumulation of flavonoids and anthocyanins in apple calli: (**A**) Phenotypes of WT and OE-156n cultured under simulated drought stress. (**B**) Flavonoid content in WT and OE-156n transgenic apple calli under simulated drought treatment. (**C**) Expression analysis of the flavonoid biosynthetic pathway genes in WT and OE-156n. Values are means SD of three independent biological replicates. Asterisks indicate statistically significant differences (* *p* < 0.05, ** *p* < 0.01, *** *p* < 0.001, and **** *p* < 0.0001, one-way ANOVA).

**Figure 7:**
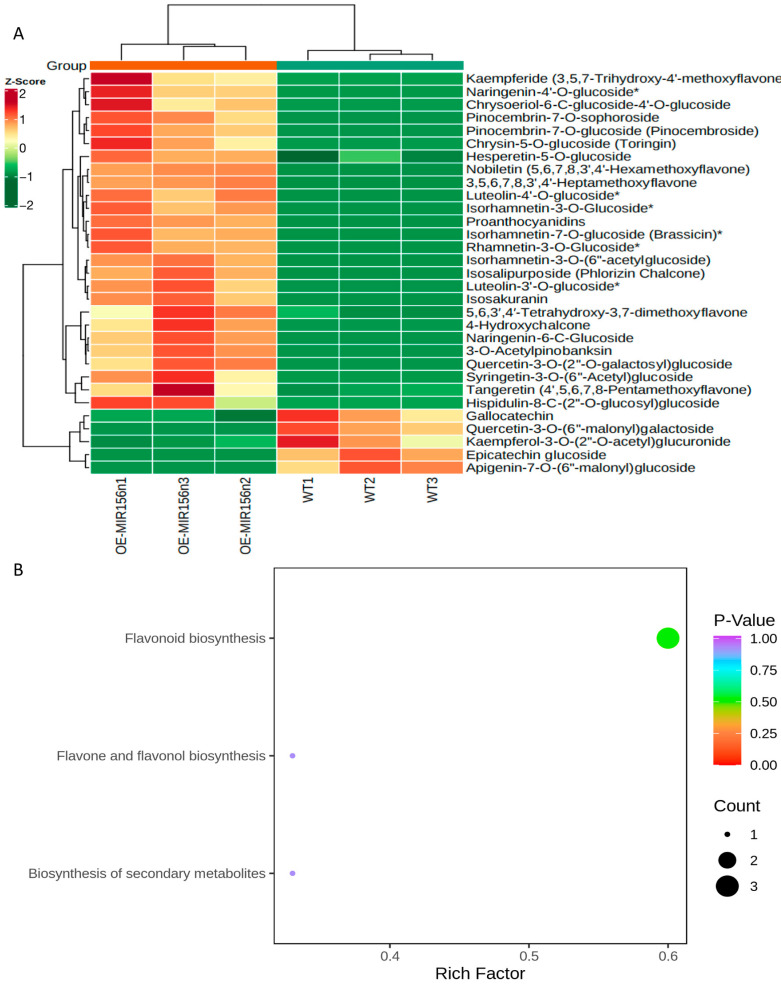
Differential flavonoid metabolite analysis in WT vs. OE-*Mdmir156n*: (**A**) Heat map of significantly differential flavonoid metabolites in WT vs. OE-*Mdmir156n*. Red and green color indicates the content of significant differential metabolites, respectively. Columns and rows represent samples and individual metabolites, respectively. The depth of color indicates the value of the correlation coefficient. The asterisk denotes the metabolite has isomerism. (**B**) Enrichment analysis of the KEGG pathway for differential metabolites in WT vs. OE-*Mdmir156n*. The color and size of the dots represents the *p* value and the amount of enriched differential metabolites, respectively. Rich factor means the ratio of the number of differential metabolites to the total number of metabolites enriched in a specific category.

**Figure 8:**
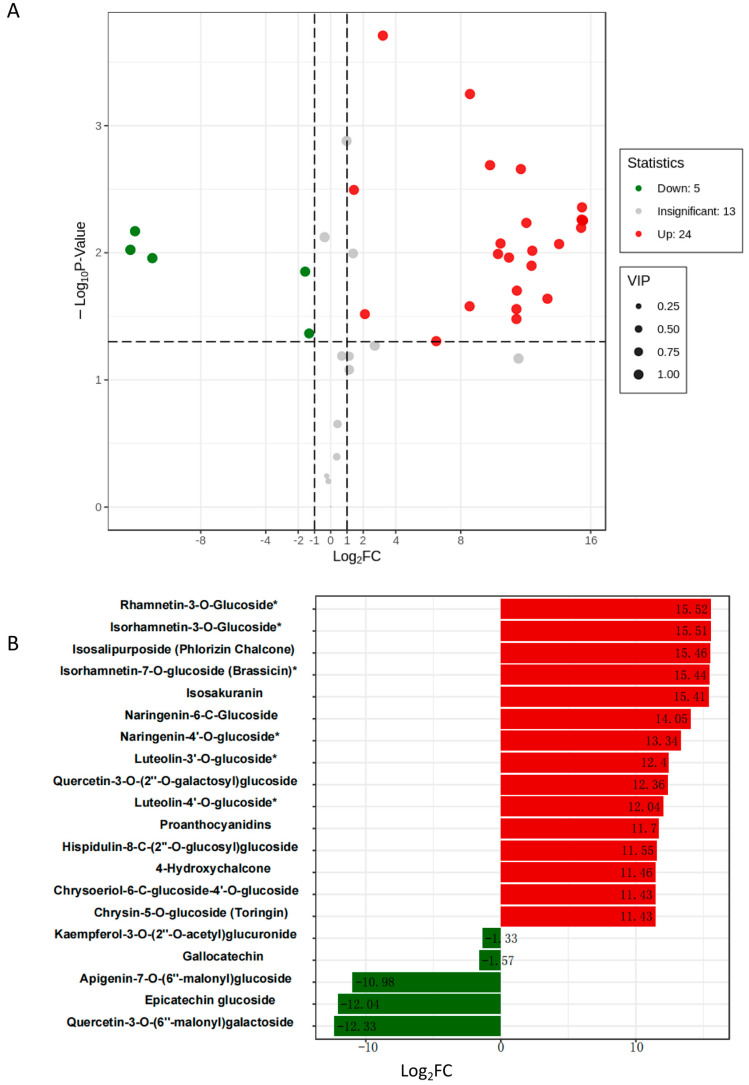
Identification of the major differential flavonoid metabolites in WT vs. OE-*Mdmir156n* apple calli (adjusted *p* < 0.05 and absolute log_2_ fold change > 10). (**A**) Volcano plot of WT vs. OE-mir156n. Green, red, and black dots represent the number of significantly downregulated, upregulated, and unchanged metabolites. (**B**) Significantly upregulated and downregulated differential flavonoid metabolites in WT vs. OE-mir156n.The asterisk denotes the metabolite has isomerism.

Consistent with the above results, after two weeks of treatment with 6%-PEG-simulating drought stress, OE-156n apple calli showed significant growth status and color difference compared to WT. The WT turned brown in color and ceased to grow. In contrast, the OE-156n apple calli appeared bright yellow and had a better growth status under the same treatment (Figure 6A). The results suggested that *MdmiR156n* might improve the growth of apple calli due to the accumulation of flavonoids under drought stress. In fact, we also measured their flavonoid content and found that the OE-156n apple calli accumulated more flavonoids than WT under drought treatment (Figure 6B). We subsequently carried out a metabolomic analysis to examine flavonoid metabolites regulated by *Mdmir156n* under drought conditions. We first applied principal component analysis (PCA) and orthogonal partial least squares discriminant analysis (OPLS-DA) on these metabolites to discover the differences within and between sample groups. The PCA and OPLS-DA results showed that the metabolites of different genotypes and treatments were significantly different and could be used for further analysis (Figure S3A,B). The normalized and transformed data of metabolites and samples in WT and OE-mir156n were presented by the clusters of heat maps (Figure 7A). A clear separation of the metabolites between ‘WT’ and ‘OE-156n’ was observed in all three replicates studied (Figure 7A). KEGG enrichment analysis suggested that the significantly differential metabolites were mainly involved in three biosynthesis pathways, namely, the flavonoid biosynthesis pathway, the flavone and flavonol biosynthesis pathway, and the biosynthesis of secondary metabolites pathway. The largest rich factor was found in the flavonoid biosynthesis pathway, which was 0.3 in WT vs. OE-miR156, followed by the flavone and flavonol biosynthesis pathway (Figure 7B). Compared to WT, there were 29 differentially accumulated flavonoid metabolites in the OE-156n apple calli. Among them, 24 were upregulated, and 5 were downregulated (Figure 8A). In particular, the levels of Rhamnetin-3-O-Glucoside, Isorhamnetin-3-O-Glucoside, and Isosalipurposide (Phlorizin Chalcone) exhibited significant upregulation in OE-mir156n than WT (Figure 8B).

In addition, the expression levels of flavonoid biosynthetic pathway genes were quantified in the OE-156n apple calli and WT after drought treatment. The qRT-PCR results suggested that the expression levels of the *MdCHS* and *MdFLS* genes were significantly higher in the OE-156n apple calli compared to WT (Figure 6C). Chalcone synthase (CHS) and flavonol synthase (FLS) are key enzymes for the production of chalcone and flavonol. On the other hand, chalcone and flavonol were the main contributors to the yellow and orange color of the plants. This is consistent with the phenotype we observed. In addition, the expression levels of anthocyanin-synthesis-related genes (DFR, ANS, and UFGF) were also significantly higher in OE-156n than in WT under the same treatment (Figure 6C). This result suggests that the overexpression of *MdmiR156n* may promote the synthesis of anthocyanidins in apple calli under drought conditions. After transferring both OE-156n and WT to a low-temperature light incubator for 10 days, we found that OE-156n actually accumulated more anthocyanins (Figure 6A).

## 3. Discussion

During the long-term struggle with environmental stress, plants have evolved a series of specific physiological and biochemical mechanisms to ensure their survival under adverse conditions [32]. In recent years, increasing evidence has demonstrated that miRNAs play critical regulatory roles in the response to various abiotic stresses. For example, the overexpression of miR1320 or miR319 enhanced the cold tolerance in transgenic rice [33,34]. miR160 significantly increased the resistance to heat in transgenic Arabidopsis [35]. miR172 is a positive regulator of salt tolerance in both rice and wheat [36]. On the contrary, overexpressed miR397 led to salt stress sensitivity being enhanced in Arabidopsis [37]. Among the many plant miRNAs, miR156 is important for plant growth and abiotic stress tolerance. Previous studies have mainly focused on its functions in plant growth and development. Recently, the function of *miRNA156/SBP* in plant abiotic stresses has gradually attracted attention. In Arabidopsis, *miRNA156* enhances plant heat tolerance by downregulating the expression of *AtSPL2,9,11* genes [38]. In apple, overexpressed miR156a enhanced the salt stress sensitivity of transgenic apple by targeting *MdSPL13*. In rice, *OsmiR156k* improved the resistance of transgenic rice to cold stress by suppressing the expression of *OsSPL3* [21]. In alfalfa, miR156 increased the tolerance of transgenic alfalfa to salt, heat, and drought stress by reducing the expression of SPL genes [15,16,17,39,40]. Consistent with previous studies, we found that the overexpression of *MdmiR56n* in Arabidopsis and apple improved the drought resistance of both the transgenic lines. With the aggravation of global warming and the destruction of ecological balance, the conflict between the environment and agricultural production is becoming more and more prominent. Drought has become one of the main factors that seriously affects the yield and quality of apple. Drought resistance is the result of various morphological, physiological, and biochemical characteristics.

To understand in depth the mechanisms of *MdmiR156* involved in drought stress tolerance, we measured various physiological and biochemical indicators associated with abiotic stresses. The root system plays a major role in plant growth and development. Changes in plant root system architecture are one of the important measures for plants to cope with abiotic stresses. Under drought stress, plants can effectively enhance drought resistance by increasing root length and changing root structure [41,42]. Recent studies have shown that miR156/SPLs regulate root growth and development in plants. In *A. thaliana*, overexpressing miR156 produce more lateral roots, whereas reducing miR156 levels leads to fewer lateral roots [43]. Further studies demonstrated that the miR156/SPL10 module is involved in plant lateral root growth through direct regulation of AGAMOUS-like MADS box protein 79 (AGL79) [8]. Furthermore, miR156/SPL10 also regulated the root meristem activity and root-derived de novo shoot regeneration via cytokinin responses [44]. Similarly, in alfalfa, miR156 significantly increased root regenerative capacity and root length by forming a miR156–SPL12–AGL6 genetic module in transgenic lines [45,46,47]. A previous finding also showed that high miR156 expression is necessary for adventitious root formation in apple [9]. Consistent with the previous results, the present study found that heterologous expression of *MdmiRl56n* significantly increased the length of primary roots and the number of lateral roots in transgenic Arabidopsis plants (Figure 2 and Figure 3). Root length and root structure are important in transporting nutrients and water from the soil and coping with abiotic stress in plants. Therefore, it is speculated that *MdmiRl56n* may improve the drought tolerance of transgenic plants by affecting their root length and structure.

In addition, cell membranes are one of the first targets of many plant stresses and it is generally accepted that the maintenance of their integrity and stability under water stress conditions is a major component of drought tolerance in plants. The degree of cell membrane injury induced by water stress may be easily estimated through measurements of electrolyte leakage from the cells. The electrolytic leakage (ET) can accurately assess the extent of cell membrane damage during drought conditions [48]. In this study, *MdmiR156n* transgenic Arabidopsis and apple lines had a lower electrolyte leakage rate and less cell membrane damage than WT after drought stress. It was shown that overexpression of *MdmiR156n* reduced cell membrane damage of transgenic lines under drought conditions. In addition, drought stress negatively affects plant growth by leading to the excessive production of reactive oxygen species (ROS) including hydrogen peroxide (H_2_O_2_), superoxide anions (O_2_^−^), hydroperoxyl radicals (·HO_2_^−^), hydroxyl radicals (·OH^−^), and alkoxy radicals [49]. Meanwhile, MDA, a major lipid peroxidation marker, is produced in large amounts after oxidative stress [50]. In order to adapt to adverse environmental conditions, plants have evolved complex and efficient antioxidant defense systems. Superoxide dismutase (SOD), the first line of protection for plant cells, can effectively scavenge ROS and protect cells from damage [51]. In fact, the SOD activity of OE-156n transgenic lines (apple and Arabidopsis) were significantly higher than the WT after drought treatment (Figure 1D and Figure 3E); in contrast, their MDA content was significantly lower than that of the WT (Figure 1D and Figure 3E). The above results indicated that OE-156n transgenic plants (apple and Arabidopsis) suffered less oxidative damage than the wild type and showed stronger drought resistance. Previous studies have shown that flavonoids can enhance plant abiotic stress tolerance by increasing SOD and POD activity to eliminate reactive oxygen species (ROS) levels [31,52]. In this study, the OE-156n apple calli appeared bright yellow and had a better growth status, while the control calli turned brown in color and ceased to grow after two weeks of drought treatment (Figure 6). Subsequently, we found many differentially accumulated flavonoid metabolites, particularly chalcones and flavonols, in OE-156n apple calli lines via metabolomic analysis, compared to the WT (Figure 8). We all know that flavonoids are the most common pigments, of which flavonols and chalcones contribute to the yellow and orange color of plants [53]. Interestingly, we also found that OE-156n apple calli lines were bright yellow under drought conditions, and their yellow color was attributed to the accumulation of chalcones and flavonols. It is well known that chalcone and flavonol are produced/catalyzed by chalcone synthase (CHS) and flavonol synthase (FLS), respectively. The qRT-PCR results suggested that the expression levels of *MdCHS* and *MdFLS* were significantly higher in the OE-156n apple calli than in WT. In addition, the flavonoid-rich OE-156n apple calli lines exhibited stronger antioxidant and drought resistance, whereas the flavonoid-deficient STTM156 lines showed the opposite phenotype (Figure 1 and Figure 6). These results showed that the overexpression of *MdmiR156n* may enhance the drought tolerance of transgenic apple by inducing the accumulation of flavonoids to promote ROS scavenging. In summary, this study provides insight into the function of *MdmiR156n* in flavonoid accumulation and drought response.

## 4. Materials and Methods

### 4.1. Plant Materials and Drought Treatments

*A. thaliana* Columbia-0 and apple ‘Orin’ calli were used for genetic transformation. *A. thaliana* plants were grown on Petri dishes with MS medium at 22 °C and under long-day conditions (8 h dark, 16 h light). The apple ‘Orin’ calli were grown on MS medium containing 0.4 mg L^−1^ 6-BA and 0.5 mg L^−1^ 2,4-D in the dark at 24 °C and were subcultured every 15 d.

Sterilized seeds from WT and T3 transgenic *A. thaliana* were sown on MS medium containing 6% PEG to simulate drought stress. After vernalization at 4 °C for 3–4 days, they were transferred to the growth chamber for normal culture. In addition, these seeds also were grown in the pot with nutrition soil and used for drought treatment by natural dehydration.

The same-weight WT and transgenic apple ‘Orin’ calli were spread on MS agar succession medium supplemented with 6% PEG to simulate drought stress in the dark at 24 °C. All experiments were repeated in triplicate.

### 4.2. Plasmid Construction, Genetic Transformation, and Generation of Transgenic Lines

The 137 bp *MdmiR156n* was amplified from genomic DNA extracted from ‘e zhen-5’ leaves as a template for PCR with a pair of gene-specific primers: 156n-F and 156n-R (Table S1). The PCR products were digested and cloned into *Xba* I and *Kpn* I sites of pCambia2300 (Clontech Laboratories, Inc., Palo Alto, CA, USA) to generate the plant overexpression vector pCambia2300-miR156n. The short tandem target mimic (STTM) technology was used to construct the STTM vector of apple miR156n as described previously [54]. The STTM miR156n sequence was designed and manually synthesized, and then was cloned into *Xba* I and *Kpn* I digested pCambia2300 plasmid. Further, the above constructed plasmids were transformed into *Agrobacterium tumefaciens* strain EHA105 and further introduced to apple ‘Orin’ calli and *A. thaliana*.

Transgenic Arabidopsis seeds were screened on MS agar medium containing 60 mg L^−1^ kanamycin for two generations (T1 and T2). T3 transgenic lines were screened on MS agar medium with 60 mg L^−1^ kanamycin and 6% PEG. The homozygous lines with drought tolerance phenotypes were selected for further study.

The transformed apple ‘Orin’ calli were spread on MS agar medium supplemented with 60 mg L^−1^ kanamycin to select positive lines. The positive lines were transferred to a new medium containing kanamycin to further remove the false positive material.

### 4.3. Quantitative Real-Time RT-PCR and Stem-Loop RT-PCR Analysis

The expression of stress-related flavonoid-related genes was measured by qRT-PCR in WT and transgenic lines of *A. thaliana* or apple calli. Total RNA was extracted from *A. thaliana* leaves or apple calli using the RNAprep Pure Plant Kit (Tiangen, Beijing, China). First-strand cDNA was synthesized using the PrimeScript™II 1st Strand cDNA Synthesis Kit (Clontech TaKaRa, Beijing, China). Quantitative RT-PCR was conducted using the ChamQ SYBR Color qPCR Master Mix Kit (Vazyme, Shanghai, China) with a QuantStudio™ 5 Real-Time PCR System. Each reaction was performed in triplicate, and data were analyzed as previously described [55]. *Atactin1* (At2g37620) or *MdActin* (XM_029088423.1) was used as an internal control. All the quantitative PCR primers are listed in Table S1.

In addition, the expression level of mature miR156 was confirmed by stem-loop RT-PCR as described [56]. The cDNA synthesis was performed using the prime script first strand cDNA Synthesis Kit (Takara, China), according to the manufacturer’s instructions, using a stem-loop RT primer instead of an oligo (dT) primer (Table S1). Subsequently, PCR was performed to detect the expression level of miR156 using the miR156-specific forward primer and the stem-loop-specific reverse primer (Table S1). The apple 5.8S rRNA (GenBank accession no. AF186480) was used as a reference gene.

### 4.4. Determination of Physiological and Biochemical Characterization of Transgenic Lines

WT and transgenic *A. thaliana* seedlings grown on the same medium were selected to measure the electrolyte leakage, as previously described [55]. After drought treatment, excised *A. thaliana* leaves and apple calli from transgenic and WT plants were stained with nitro blue tetrazolium (NBT) to detect O_2_^−^ accumulation as described previously [55]. In addition, the activity of superoxide dismutase (SOD) and malondialdehyde (MDA) content was measured using the assay kit following the manufacturer’s instructions (Solarbio, Beijing, China).

### 4.5. Extraction and Determination of Total Anthocyanin and Flavonoid Content

After one week of natural drought for 3-week-old *A. thaliana* (WT and transgenic plants) grown in the same pot, the leaves were taken to extract anthocyanin using 1% (*v*/*v*) HCl-methanol extraction solution at 4 °C in the dark for 24 h, to determine the anthocyanin content using the pH difference method according to the previous method [57].

The apple calli treated with 6% PEG for two weeks were taken to extract flavonoids by incubating in 1% (*v*/*v*) HCl methanol for 4 h at 4 °C, to measure the flavonoid content using an ultra-sensitive multi-function microchannel plate detector (Biotek, Cytation 1, Winooski, VT, USA). Rutin (Yuanye Bio-Technology, Shanghai, China) was used as the master standard. 

### 4.6. Metabolite Extraction and UPLC-ESI-MS Analysis of WT and OE-156n Apple Calli

UPLC-MS analysis was performed according to the previous methods [58], with modifications: dissolve 100 mg of lyophilized powder of WT and OE-156n apple calli with 1.2 mL 70% methanol solution, vortex 30 s every 30 min for 6 times in total, then place the sample in a refrigerator at 4 °C overnight. Following centrifugation at 12,000 rpm for 10 min, the extracts were filtrated (SCAA-104, 0.22 μm pore size; ANPEL, Shanghai, China, http://www.anpel.com.cn/, accessed on 8 September 2021). The filtrated extracts were analyzed using an UPLC-ESI-MS/MS system (UPLC, SHIMADZU Nexera X2, Kyoto, Japan; MS, Applied Biosystems 4500 Q TRAP, Woodlands, Singapore). The analytical conditions refer to previous descriptions [59]. The effluent was alternatively connected to an ESI-triple quadrupole-linear ion trap (QTRAP)-MS. The ESI source operation parameters were as follows: ion source, turbo spray; source temperature, 550 °C; ion spray voltage (IS), 5500 V (positive ion mode)/−4500 V (negative ion mode); ion source gas I (GSI), gas II (GSII), and curtain gas (CUR) were set at 50, 60, and 25.0 psi, respectively; the collision-activated dissociation (CAD) was high. Instrument tuning and mass calibration were performed with 10 and 100 μmol/L polypropylene glycol solutions in QQQ and LIT modes, respectively. QQQ scans were acquired as MRM experiments with collision gas (nitrogen) set to medium. DP and CE for individual MRM transitions was conducted with further DP and CE optimization. A specific set of MRM transitions were monitored for each period according to the metabolites eluted within this period.

### 4.7. Statistical Analysis

The data in this study are expressed as the ± SD of three independent biological replicates unless otherwise indicated. A one-way ANOVA analysis was used to calculate the significance of differences.

Significantly regulated metabolites between groups were determined by VIP ≥ 1 and absolute log_2_FC (fold change) ≥2. VIP values were extracted from the OPLS-DA result, which also contains score plots and permutation plots, and was generated using R package MetaboAnalystR. The data was log transformed (log_2_) and mean centering before OPLS-DA. In order to avoid overfitting, a permutation test (200 permutations) was performed. Identified metabolites were annotated using the KEGG Compound database (http://www.kegg.jp/kegg/compound/, accessed on 8 September 2021); annotated metabolites were then mapped to the KEGG Pathway database (http://www.kegg.jp/kegg/pathway.html, accessed on 8 September 2021). Pathways with significantly regulated metabolites mapped to were then fed into MSEA (metabolite sets enrichment analysis), and their significance was determined by hypergeometric test *p*-values.

## Data Availability

The data presented in this study are available in the article and the supplementary materials here.

## Supplementary Materials

The following supporting information can be downloaded at https://www.mdpi.com/article/10.3390/ijms24076049/s1.

## Abbreviations

MS	Murashige and Skoog
PEG	polyethylene glycol
O_2_^−^	superoxide anion radicals
MDA	malondialdehyde
SOD	superoxide dismutase
ROS	reactive oxygen species
ET	electrolytic leakage
H_2_O_2_	hydrogen peroxide
HO_2_^−^	hydroperoxyl radicals
OH^−^	hydroxyl radicals
STTM156	Short Tandem Target Mimic 156
WT	wild type
ROS	reactive oxygen species
NBT	nitro blue tetrazolium
CHS	chalcone synthase
FLS	flavonol synthase
