# Supplementary material for: The MdmiR156n Regulates Drought Tolerance and Flavonoid Synthesis in Apple Calli and Arabidopsis
Source: Int J Mol Sci. 2023 Mar 23;24(7):6049. doi: 10.3390/ijms24076049 (PMC10094179; doi:10.3390/ijms24076049)
Supplement: Supplementary file 1 [file ijms-24-06049-s001.zip › ijms-2262857-supplementary.pdf]

The *MdmiR156n* Regulates Drought Tolerance and Flavonoid Synthesis in Apple Calli and *Arabidopsis*

Guo Chen [1,2,†], Yaping Wang [1,2,†], Xueli Liu [1,2], Siyue Duan [1,2], Shenghui Jiang [1,2], Jun Zhu [1,2], Yugang Zhang [1,2,*] and Hongmin Hou [1,2*]

[1] College of Horticulture, Qingdao Agricultural University, Qingdao 266109, China
[2] Engineering Laboratory of Genetic Improvement of Horticultural Crops of Shandong Province, Qingdao Agricultural University, Qingdao 266109, China
* Correspondence: ygzhang@qau.edu.cn (Y.Z.); hmhou@qau.edu.cn (H.H.); Tel.: +86-0532-860-80752 (H.H.)
† These authors contributed equally to this work.

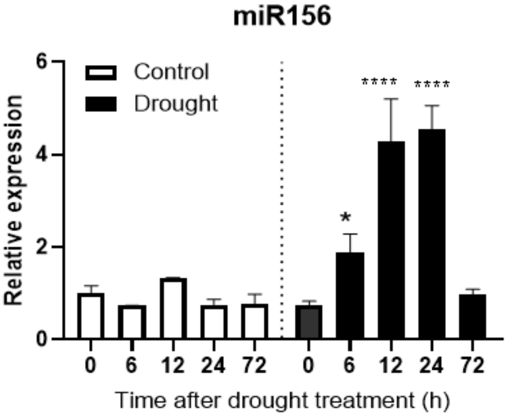

Figure S1. Response of *MdmiR156* to drought stress in apple. Asterisks indicate statistically significant differences (* $p < 0.05$ and **** $p < 0.0001$, one-way ANOVA).

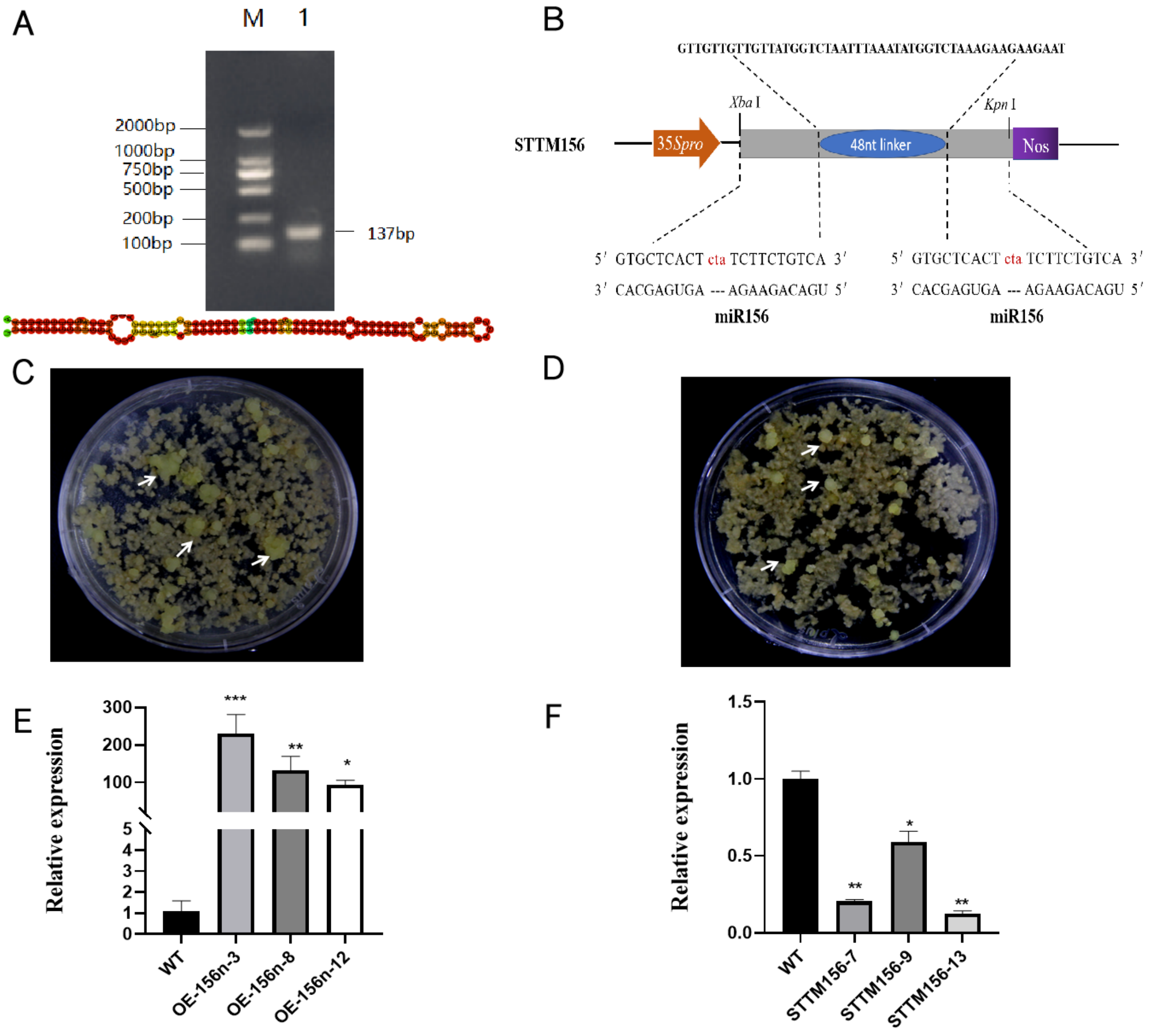

Figure S2. Screening and identification of the OE-156n and STTM-156n transgenic resistant apple 'Orin' callis. (A) Cloning of *MdmiR156n* precursor genes. (B) The diagram of STTM-miR156 structure with 48 nt imperfect stem-loop. (C, D) Screening the OE-156n and STTM-156n transgenic resistant apple 'Orin' callis with kanamycin. The arrows indicate newly generated callis. (E, F) Identification of the OE-156n and STTM-156n transgene positive lines by qRT-PCR. Values are means SD of three independent biological replicates. Asterisks indicate statistically significant differences (* $p < 0.05$, ** $p < 0.01$, *** $p < 0.001$, one-way ANOVA).

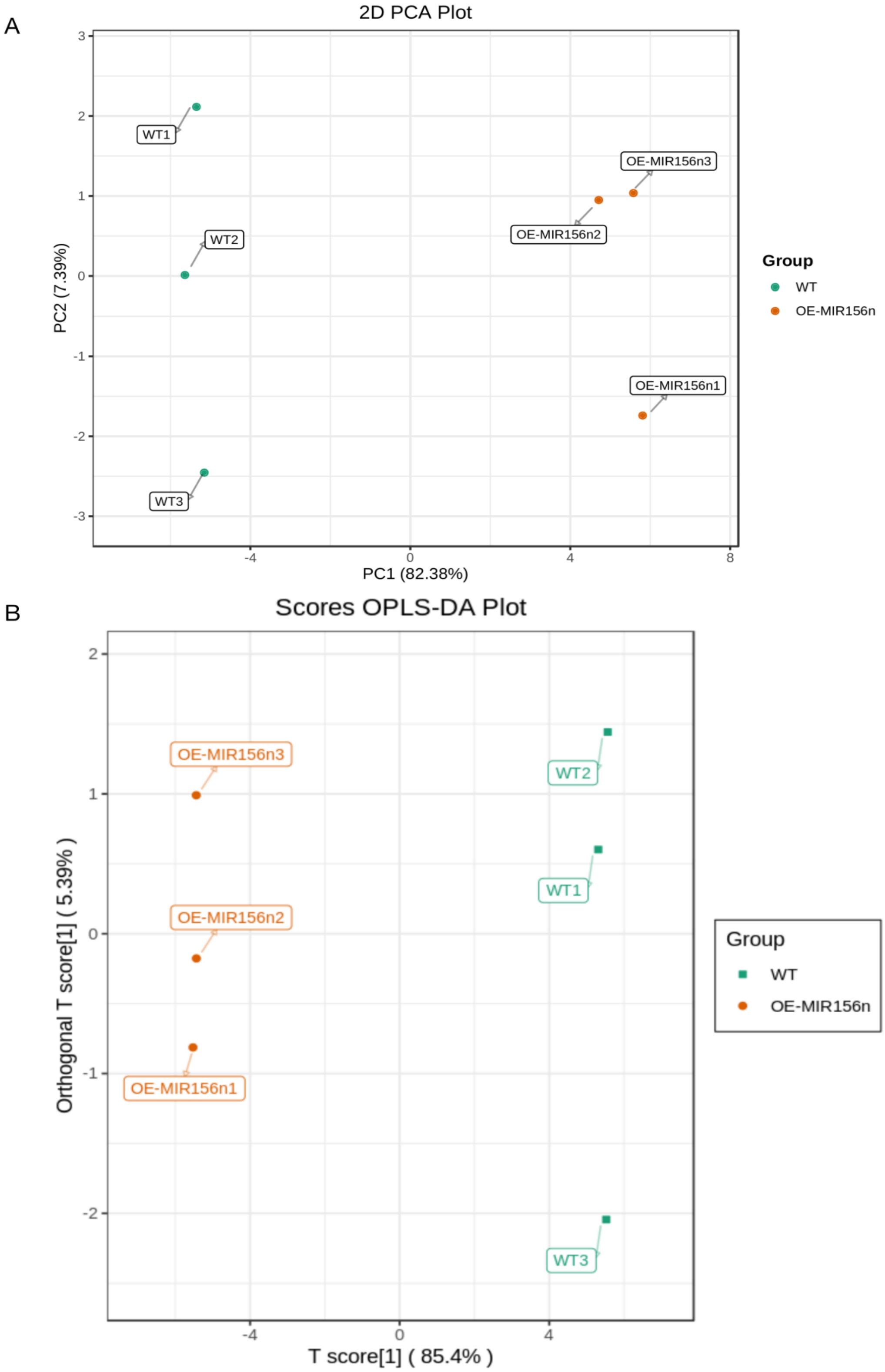

Figure S3. Quality control of metabolomics analysis of WT and OE-MIR156n lines under drought stress. A. PCA analysis. B. OPLS-DA analysis.

Table S1. The information of primers used in this paper. F indicates forward primer, R indicates reversed primer and the underline indicates restriction sites.

Primer Name	Sequence (5′-3′)
MdActin-F	ATTCAAGTATGCCTGGGTGC
MdActin-R	CAGTCAGCCTGTGATGTTCC
Atactin1 -F	AGGCACCTCTTAACCCTAAAGC
Atactin1 -R	GGACAACGGAATCTCTCAGC
MdNCED3-F	CCCGACTGCTTCTGCTTCCA
MdNCED3-R	AGCCGGATTTCTGACAAGACG
MdDREB2-F	ATGGCCTATGACGATGCTGC
MdDREB2-R	GAAGTTCCAAATGGAAGTGG
MdP5CS1-F	AAGTCGATGTCAGTTGTGG
MdP5CS1-R	ATAAATGTGAGATGTCCAAGCG
MdRD22-F	CACGTGGACTTTCGGATC
MdRD22-R	CTCCTGCTCCAGTGGAC
AtNCED3-F	TTGATGCTCCAGATTGCTTC
AtNCED3-R	GGACCCTATCACGACGACTT
AtP5CS1-F	TTCTCAGATGGTTTCCAGGTTG
AtP5CS1-R	TGGGAATGTCCTGATGGGTG
AtRD29B-F	GTGAAGATGACTATCTCGGTGGTC
AtRD29B-R	TACCAAGAGACTCAGCAATCTCTG
AtPAL1-F	CTTGGAACAGAGCTTTTGACCG
AtPAL1-R	CGTGAAAACCTTGTCGAACTCTTC
AtCHS-F	GGAGAAGTTCAAGCGCATGTG
AtCHS-R	ATGTGACGTTTCCGAATTGTCG
AtCHI-F	CTCTCTTACGGTTGCGTTTTCG
AtCHI-R	CACCGTTCTTCCCGATGATAGA
AtFLS1-F	CCACCGTCATGCGTCAATTACAG
AtFLS1-R	TCTCCGCCGAGACCTTCTTTCAA
AtFLS3-F	GCTTGTGGCAAGTGCGGTGG
AtFLS3-R	GCTCGGTCGGGATTCCGTGG
AtDFR-F	AGCCGCCAAGGGACGTTATATTTG
AtDFR-R	CCGGGAGAAAACCCTTTTGACGA
AtF3′H-F	TTCCTTACCTTCAGGCGGTTATC
AtF3′H-R	CGAGAGTGGTGTTGGTGGATG
AtANS-F	GGCTGTGTTTTGTGAGCCACCA
AtANS-R	CCTTGGAGGAAACTTAGCCGGAGA
AtUGT78D2-F	TTTTGGCGCATTGTGCTGT
AtUGT78D2-R	TCAGCAAACTGCGGAAACG
AtUGT75C1-F	CGGTGTTGGAGAGTGTATCGG
AtUGT75C1-R	TGATCCCCAAAAAATGGCC
MdPAL-F	ACCCTGGACAGATTGAGGCAGCT
MdPAL-R	GCGTAGCGATCCTGCTTTGGCT
MdCHS-F	GTGACTGTCCAGGAAGTTCGC
MdCHS-R	GCACACACTTGGATTCTCCTTTAG
MdCHI-F	GAAGGGTAAGACCGCCGAG
MdCHI-R	CACAATTCTCCGAAACTTTCTCAG
MdF3H-F	CGGGATGATGGGAAAACG
MdF3H-R	CGCTGGGTTCTGGAATGTG
MdF3′H-F	ACGATGGCGGATGTTACGG
MdF3′H-R	GCTTTGACCCTGCACTTGCT
MdDFR-F	GGACCCCGAGAATGAAGTG

MdDFR-R	CTCCACATTCACGGTTCCTG
MdANS-F	GAGAAGTATGCCAATGACCAGG
MdANS-R	GGCGGTTGCCTCAATGTAAT
MdUFGT-F	GCTGACGAGTTGGGAGTGC
MdUFGT-R	CCTTCCGCTAAGTCTTTGATTC
MdFLS-F	ACGAGCAACCGGGAATCACAACTG
MdFLS-R	CCCAGTTGGAGCTGGCCTCAGTA
35S-F	AGATAGTGGAAAAGGAAGGTGGC
156n-F	TCTAGATTAATCTGGTTGGACTTAGGGTAG
156n-R	GGTACCTTAATCCGGCAGTGGAAA
Md-5.8S rRNA-F	GCAACGGATATCTCGGCTCT
Md-5.8S rRNA-R	CAACTTGCGTTCAAAGACTCG
35S-F	AGATAGTGGAAAAGGAAGGTGGC
STTM156n-R:	CTGACAGAAGACTAGAGTGAGC
Reverse transcription primer	GTCACATCGTATCGTGAAGCTGCGCAGCTGATGTGACGTGCTCAC
Universal PCR reverse primer	CACATCGTATCGTGAAGCTGC
MdmiR156-F	TGCACTAGCGTGTGACAGAAGAGA
